# Predictors of health status do not change over three-year periods and exacerbation makes difference in chronic obstructive pulmonary disease

**DOI:** 10.1186/1477-7525-9-112

**Published:** 2011-12-09

**Authors:** Renata Ferrari, Suzana E Tanni, Laura MO Caram, Cristiane R Naves, Irma Godoy

**Affiliations:** 1 Faculdade de Medicina, UNESP-Univ Estadual Paulista, Campus de Botucatu, Departamento de Clínica Médica, Botucatu, SP, Brasil

**Keywords:** COPD, Health status, BODE index, dyspnea

## Abstract

**Background:**

The association between disease markers and health status (HS) overtime is unclear. The aim of this study was to verify the predictors of HS at baseline and after three years in Chronic Obstructive Pulmonary Disease (COPD) patients.

**Methods:**

Ninety-five consecutive COPD patients (66% male, age = 67 ± 9 y, FEV_1 _= 58 ± 23%) underwent the following evaluations at baseline and after three years: body composition, pulse oximetry (SpO_2_), six-minute walk distance (6MWD), Modified edical Research Council dyspnea scale (MMRC) and Saint George's Respiratory Questionnaire (SGRQ). The Charlson comorbidity index and BODE index were calculated. COPD exacerbations during the follow-up were evaluated. At baseline, age, gender, smoking, SpO_2_, BODE index or its components (BMI, MMRC, FEV_1 _and 6MWD), and Charlson index were included in a multiple linear regression analysis with the baseline SGRQ total score as the dependent variable. After three years, we included the final values of the variables plus the number of exacerbations and the final SGRQ total score as the dependent variable.

**Results:**

SGRQ total score (42 ± 19% *vs *44 ± 19%; p = 0.041) and activity domain (52 ± 21% *vs *60 ± 22%; p < 0.001) deteriorated during follow-up. At baseline, BODE index was selected as a predictor of SGRQ total score (R^2 ^= 0.46; p < 0.001); after three years, BODE index and age were the predictors (R^2 ^= 0.49; p < 0.001). When the BODE index was replaced by its variables, MMRC was selected as the only variable associated with the SGRQ total score (R^2 ^= 0.58; p < 0.001). After three years, MMRC, FEV_1 _and number of exacerbations were selected as predictors of SGRQ total score (R^2 ^= 0.63; p < 0.001).

**Conclusion:**

HS deteriorated significantly over the three-year period and the predictors of HS do not change over time. BODE index and dyspnea were predictors at baseline and after three years. Exacerbation was also a predictor of HS after three years.

**Trial Registration:**

ClinicalTrials.gov:  NCT00605540

## Introduction

Chronic obstructive pulmonary disease (COPD) has significant extrapulmonary consequences that lead to comorbidity conditions and effects on patients' quality of life (QoL) [1]. Jones [2] empathizes that it is important to make a distinction between QoL and and health status (HS) measurement, since QoL has become a central feature of studies in COPD and its impairment reflects the impact of disease in the patient. While HS measurement is a standardized quantification of the impact of the disease. The purpose of these measurements is to address a wide range of effects of the disease, thus provide emotional and psychological aspects of the illness as well as the physical; however the most of their items usually concern practical aspects of disturbance to daily life [3].

Health status is an important measurable outcome in patients with COPD, since it is identified as a predictor of mortality and often worsens significantly with disease progression [4-7]. Dyspnea perception, nutritional depletion, exercise tolerance impairment, exacerbation frequency, and the BODE index have been identified as predictors of HS. However, in the best equations, these predictors explain 25% to 46% of the HS differences between patients with COPD [7-11]. In addition, only two studies verified associations between modifications of disease markers and HS and both did not include exacerbation rate as a predictor over time [7,11]. Exacerbations of COPD indicate progression of the disease and are associated with reduced health status [12]. Therefore, we hypothesized that the rate of exacerbation may be influential in the health status over time. Identification of predictors of HS overtime may open a window of opportunity to direct resources in disease management. Thus, the aim of this study was to verify the predictors of health status at baseline and after three years in COPD patients.

## Methods

### Patients

In a prospective study were recruited one hundred and thirty three consecutive COPD patients with mild to very severe COPD from the outpatient clinic of a single institution. Major inclusion criteria were clinical diagnosis of COPD according to criteria set out in GOLD 2009 and the Brazilian Thoracic Society (BTS) [1,13], age ≥ 40 years, smoking history ≥ 10 pack-years, and a post-bronchodilator FEV_1_/FVC ratio < 70%. Disease severity was categorized according of BTS and GOLD stages taking in consideration the values of FEV_1 _(% predicted) and arterial blood gases (GOLD I: FEV_1 _≥ 80%; GOLD II: 50 ≤ FEV_1 _< 80%; GOLD III: 30 ≤ FEV_1 _< 50%; GOLD IV: FEV_1 _< 30% or < 50% plus chronic respiratory failure). The following factors were considered grounds for exclusion: a history of asthma and/or FEV_1 _increased > 12% or 200 mL post-bronchodilator test, associated restrictive disorder (tuberculosis sequelae, interstitial fibrosis); other clinically significant concomitant respiratory diseases (sleep apnea/hypopnea syndrome, lung cancer); noncompliance with COPD treatment; myocardial infarction within the preceding four months; and unstable angina or congestive heart failure (New York Heart Association class III or IV). Patients not considered clinically stable (i.e., with changes in medication dose or frequency, disease exacerbation, or hospital admissions in the preceding 6 weeks) were also excluded. All patients were optimized in terms of standard medical therapy according to GOLD and BTS guidelines [1,13]. Active smoking patients received practical advice to quit smoking and were referred to smoking cessation program. Patients with chronic hypoxemia received a stable dose of oxygen therapy over the 6 months before study enrollment.

Participants were made aware of the proposed study procedures and freely gave written informed consent. All procedures were approved by the Research Ethics Committee, Botucatu Medical School University Hospital (390/2007-CEP).

### Measurements

Spirometry was performed, using the KOKO Spirometer, before and 15 minutes after the inhalation of 400 mcg salbutamol (Ferrari KOKO Louisville, CO 80027, USA), according to criteria set by the American Thoracic Society [14]. FEV_1 _values are expressed in liters, percentages of FVC, and percentages of reference values [15]. Pulse oximetry (SpO_2_) was assessed using a Onyx oxymeter (Model 9500 Oximeter; Nonin Medical Inc.; Minneapolis, MN, USA) while patients were breathing room air. Body weight and height were measured. Body mass index [BMI = weight in kg/(height in m)^2^] was calculated. Smoking history was obtained by patient interview using standardized instruments at baseline and smoking cessation by self report during patients' contacts. A translated version of the Saint George's Respiratory Questionnaire (SGRQ), validated for use in Brazil, was utilized to evaluate patient HS [16]. Minimum clinically important difference (MCID) was defined as a decrease of ≥ 4% in the SGRQ domains [17]. Dyspnea was assessed using a translated version of the Modified Medical Research Council (MMRC) scale [18]. The six-minute walk distance (6MWD) was performed according to American Thoracic Society guidelines [19]. BMI/airflow obstruction/dyspnea/exercise capacity (BODE) index was calculated using the model described by Celli et al. [20] BODE scores were categorized as class 1 (score: 0 to 2), class 2 (score: 3 to 4); class 3 (score: 5 to 6); and class 4 (score: 7 to 10) [20]. Comorbid disease data were collected from patient medical records and quantified according to the Charlson index [21]. Patients or family, in the case of death, were contacted by telephone every 3 months to determine the occurrence of exacerbations or hospital admissions. During the telephone interview a structured questionnaire was used to identify data associated with exacerbation and/or hospitalizations. Data were confirmed during clinic visits and by reviewing medical records. An exacerbation was defined as an increase in dyspnea, sputum purulence, and increased sputum volume and classified as moderate (requiring a visit to a doctor or the emergency department and treatment with antibiotics or systemic steroids or both) or severe type II (requiring hospital admission) [22]. Mild exacerbations not requiring intervention were not included in the study.

### Statistical analysis

All data were analyzed using SigmaStat 3.2 (Inc, Chicago, IL, USA) and STATA 10.0 (Stata Corp, Texas, USA). Mean ± SD or median interquartile range (25-75%) was used depending on distribution. Paired t-test or Wilcoxon test was performed to compare characteristics at baseline to those presenting after three years. At baseline, age, gender, smoking status, SpO_2_, BODE index or its components (BMI, MMRC, FEV_1 _and 6MWD), and Charlson index were included in a multiple linear regression analysis with the baseline SGRQ total score as the dependent variable. This analysis was done separately for all patients evaluated at baseline and for those followed during three years. After three years, we included the final values of the same variables with the final SGRQ total score as the dependent variable. In another model, we evaluated the influence of the number of exacerbations in the previous model. This variable was included only in the final moment because reliable information on exacerbations was not available at baseline and was collected during the follow-up period. The variables included were those known to be associated with HS in the literature and the potential confounders [7-11]. Age and gender at baseline and the difference between baseline and after 3 years measurements (Δ) for pulse oximetry (Δ SpO_2_), Δ Bode index, Δ Charlson index and number of exacerbation were included in a multiple logistic regression to evaluate the influence of these variables on clinically significant stability/improvement or worsening, defined as a change ≥ 4%, of the SGRQ domains. We repeated the previous analyses replacing the BODE index by its components. A p < 0.05 was defined as statistically significant.

## Results

The baseline characteristics of the 133 patients (69% men) were mean age of 65 ± 9 years and smoking exposure of 53 ± 28 pack-years; 45 patients (34%) were active smokers. Seventy-two patients were using long-term broncodilators and 49 patients were regularly using inhaled corticosteroid, 25 had been on stable oxygen flow therapy for the last six months. No patients were medicated with theophylline or leukotriene modifiers. A total of 3 (2%) patients presented congestive heart failure class I or II, 6 (4%) patients presented dyslipidemia, 9 (6%) patients presented diabetes mellitus and 42 (31%) patients presented arterial hypertension at baseline.

**Figure 1:**
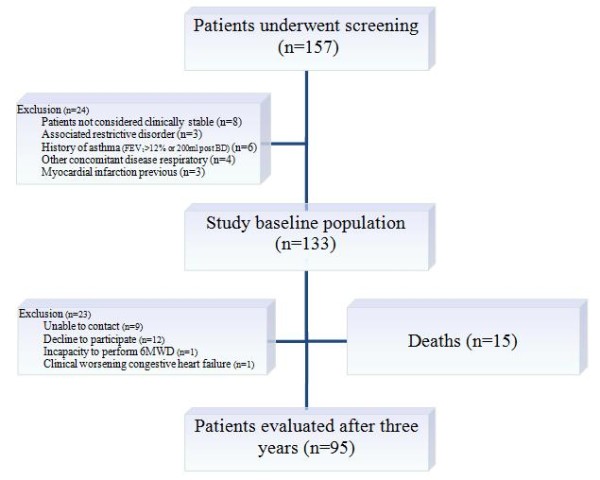
**Diagram of patient follow up in three-year period**.

Of the 133 patients initially evaluated, 38 were excluded from the final analyses; 15 patients died and 23 dropped out. Thus, 95 patients were monitored for three years (Figure 1). Comparisons of the excluded patients versus those completing the study did not show significant differences at baseline (data not shown).

At baseline, the mean age of the 95 studied patients (66% men) was 64 ± 9 years and smoking exposure was 54 ± 28 pack-years; 32 patients (33%) were active smokers, and 8 of them stopped smoking during follow-up. The comparison of patient characteristics between baseline and after three years is shown in Table 1 and has been presented in a previous publication [23].

**Table 1:** Characteristics of COPD patients followed-up over a three-year period

Variables	Initial Assessment (n = 95)	Final Assessment (n = 95)	p-value
FEV_1 _(%)	59.3 ± 23.2	58.5 ± 22.7	0.228
FEV_1 _(L)	1.4 ± 0.6	1.3 ± 0.5	**< 0.001**
FVC (%)	90.8 ± 23.8	88.9 ± 24.7	0.167
FVC (L)	2.7 ± 0.8	2.5 ± 0.8	**0.004**
FEV_1_/FVC	52.2 ± 11.7	51.3 ± 10.4	0.123
BMI (kg/m^2^)	25.9 ± 5.8	25.8 ± 5.6	0.382
SpO_2 _(%)	93.6 ± 3.1	92.0 ± 4.8	**< 0.001**
MMRC (score)	1.5 ± 1.0	1.9 ± 1.1	**0.002**
6MWD (m)	437.7 ± 85.6	412. 4 ± 100.0	**0.001**
Charlson index (score)	3.5 ± 1.5	3.9 ± 1.4	**0.009**
BODE index (score)	2.2 ± 1.8	2.6 ± 2.3	**0.008**

Paired t-test or Wilcoxon. Values are presented as mean ± SD or as median (25-75% interquartile range). FEV_1_: forced expiratory volume in the first second (% of predicted); FVC: forced vital capacity (% of predicted); BMI: body mass index; SpO_2_: pulse oximetry; MMRC: Modified Medical Research Council; 6MWD: six-minute walking distance; p < 0.05.

At baseline, 18% of patients were in GOLD stage I, 39% were in stage II, 19% were in stage III, and 24% were in stage IV COPD. There was no difference in the proportion of patients within each disease severity between baseline and after three years (p = 0.865). According to BODE index [20], at baseline, 57 were in class 1, 21 in class 2 and 17 were in class 3. After three years, there was significant different between the classes, since 51 were in class 1, 23 in class 2, 14 in class 3 and 7 patients in class 4 (p < 0.05).

**Figure 2:**
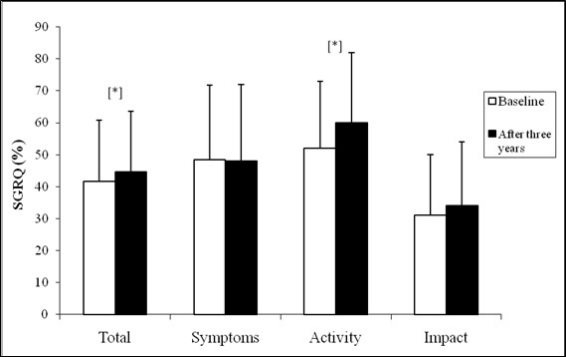
**Mean SGRQ domains at baseline and after three years**. SGRQ: Saint George's Respiratory Questionnaire; *p < 0.05.

Health status showed significant worsening in the activity domain score (52 ± 21 vs. 60 ± 22%, p < 0.001) and SGRQ total score (42 ± 19 vs. 44 ± 19%, p = 0.041) (Figure 2). The SGRQ total scores were significantly higher for patients in stage IV than for patients in stages I and II, and also for patients in stage III than for patients in stage I and for patients in stage II than patients in stage I. We did not identify differences between stages II and III and stages III and IV after three years. In the BODE classification, we found that HS change between the classes 1 and 2, classes 1 and 3 and classes 1 and 4 after three years.

Seventy-two patients (75.8%) had at least one exacerbation during the study period and in these patients the baseline SGRQ total score was significantly higher [44 (30-61)%] in those without exacerbation [27 (14-39)%, p < 0.001].

In the multiple linear regression analysis, the BODE index was selected as predictor of SGRQ total score at baseline (R^2 ^= 0.46; p < 0.001). After three years, the BODE index and the patient age were the predictors in the model without exacerbation (R^2 ^= 0.49; p < 0.001) (Table 2).When exacerbation was included, the variables selected did not change (R^2 ^= 0.51; p < 0.001) (data not shown). When BODE index was replaced by its variables (BMI, MMRC, FEV_1 _and 6MWD), MMRC was the predictor of SGRQ total score at baseline (R^2 ^= 0.58; p < 0.001) and MMRC and FEV_1 _after three years (R^2 ^= 0.61; p < 0.001) (Table 3). When number of exacerbations was included in the model, the predictors of HS were MMRC, FEV_1 _and exacerbation (R^2 ^= 0.63; p < 0.001) (Table 4). At baseline, predictors of HS for 133 patients were the same shown for 95 patients followed during three years, BODE index and the patient age (data not shown). Simple correlation analysis between baseline and final SGRQ score and age, gender, smoking, SpO_2_, BODE index or its components, and Charlson index are included as additional file 1.

**Table 2:** Multiple linear regression model to evaluate predictors for baseline total SGRQ and after three years follow-up (n = 95)

Variables	Baseline total SGRQ Coefficient (95% CI)	p-value	Final total SGRQ Coefficient (95% CI)	p-value
Male	-3.41 (-11.09, 4.27)	0.380	-0.42 (-7.59, 6.73)	0.905
Age (years)	-0.52 (-1.08, 0.03)	0.063	-0.62 (-1.13, -0.10)	**0, 019**
Smoking status	1.68 (-5.83, 9.20)	0.658	-2.70 (-10.21, 4.75)	0.470
SpO_2 _(%)	-0.60 (-1.72, 0.52)	0.291	-0.35 (-1.06, 0.35)	0.326
Bode index (score)	5.59 (3.73, 7.45)	**< 0.001**	4.90 (3.41, 6.40)	**< 0.001**
Charlson index (score)	-1.54 (-4.46, 1.36)	0.294	0.29 (-2.60, 3.20)	0.840

SGRQ: SpO_2_: pulse oximetry; Baseline (R^2 ^= 0.46; p < 0.05); After three years (R^2 ^= 0.49; p < 0.05)

**Table 3:** Multiple linear regression model to evaluate predictors for baseline total SGRQ and after three years follow-up (n = 95)

Variables	Baseline total SGRQ Coefficient (95% CI)	p-value	Final total SGRQ Coefficient (95% CI)	p-value
Male	1.11 (-6.32, 8.56)	0.766	-1.02 (-7.67, 5.62)	0.760
Age (y)	-0.51 (-1.05, 0.01)	0.059	-0.14 (-0.67, 0.37)	0.570
Smoking	5.68 (-1.55, 12.93)	0.122	1.96 (-5.46, 9.40)	0.600
SpO_2 _(%)	-0.22 (-1.29, 0.84)	0.675	-0.27 (-0.91, 0.36)	0.395
FEV_1 _(%)	-0.11 (-0.25, 0.25)	0.105	-0.18 (-0.32, -0.05)	**0.007**
6MWD (m)	-0.01 (-0.05, 0.03)	0.731	0.01 (-0.02, 0.56)	0.426
BMI (kg/m^2^)	-0.24 (-0.75, 0.23)	0.349	0.18 (-0.34, 0.72)	0.489
MMRC (score)	11.72 (8.17, 15.26)	**< 0.001**	10.44 (7.08, 13.80)	**< 0.001**
Charlson index	-1.16 (-3.82, 1.48)	0.384	-0.39 (-3.10, 2.30)	0.770

SpO_2_: pulse oximetry; FEV_1_: forced expiratory volume in the first second (% of predicted); 6MWD: six-minute walking distance; BMI: body mass index; MMRC: Modified Medical Research Council; Baseline (R^2 ^= 0.58; p < 0.05); After three years (R^2 ^= 0.61; p < 0.05).

**Table 4:** Multiple linear regression model to evaluate predictors for total SGRQ after three years follow-up (n = 95)

Final total SGRQ (%)	Dependent variables	Coefficient (95% CI)	p-value
	Male	-0.14 (-6.69, 6.40)	0.965
	Age (y)	-0.16 (-0.67, 0.34)	0.525
	Smoking	3.05 (-4.28, 10.39)	0.410
	SpO_2 _(%)	-0.27 (-0.89, 0.35)	0.389
	FEV_1 _(%)	-0.14 (-0.28, -0.01)	**0.043**
	6MWD (m)	0.01 (-0.02, 0.05)	0.433
	BMI (kg/m^2^)	0.27 (-0.26, 0.79)	0.315
	MMRC (score)	9.99 (6.68, 13.30)	**< 0.001**
	Charlson index (score)	-0.19 (-2.84, 2.45)	0.883
	Number of exacerbations	1.29 (0.11, 2.47)	**0.031**

SpO_2_: pulse oximetry; FEV_1_: forced expiratory volume in the first second (% of predicted); 6MWD: six-minute walking distance; BMI: body mass index; MRC: Modified Medical Research Council; R^2 ^= 0.63; p < 0.05.

**Figure 3:**
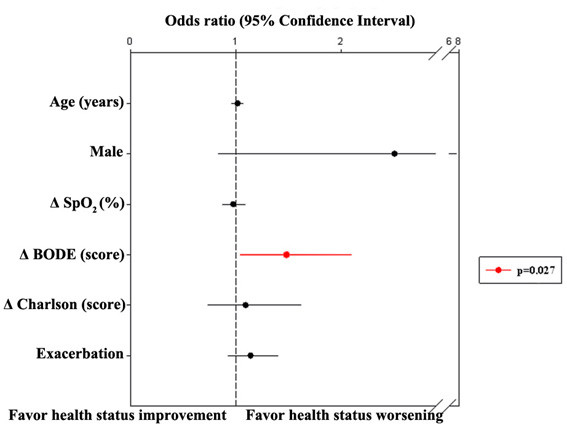
**Multiple logistic regression analysis to evaluate the predictors for stability/improvement or worsening (≥ 4%) on SGRQ total score (n = 95)**. Δ: final assessment values-initial assessment values; SpO_2_: pulse oximetry; Exacerbation: number of exacerbations for patient in the three-year period.

**Figure 4:**
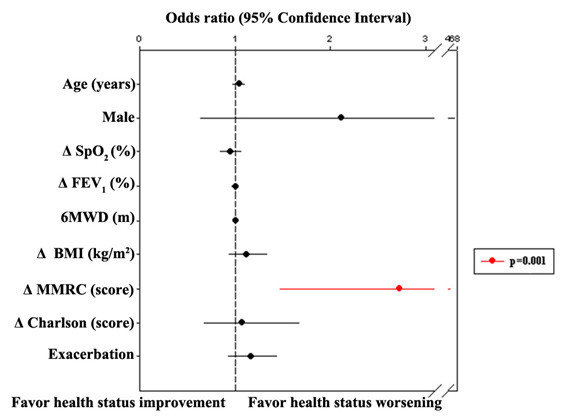
**Multiple logistic regression analysis to evaluate the predictors for stability/improvement or worsening (≥ 4%) on SGRQ total score (n = 95)**. Δ: final assessment values-initial assessment values; SpO_2_: pulse oximetry; FEV_1_: forced expiratory volume in the first second (% of predicted); 6MWD: six-minute walking distance; BMI: body mass index; MMRC: Modified Medical Research Council; Exacerbation: number of exacerbations for patient in the three-year period.

Fifty-one percent of the patients presented with clinical worsening (≥ 4%) on SGRQ total score, and 59% of them were in severe to very severe stages of the disease. A total of 28% reported clinical improvement and 21% had no clinical change on SGRQ total score. In the multiple logistic regression analysis, modification in the BODE index was the predictor of clinically significant worsening on SGRQ total score [OR 1.48 (95% CI 1.04-2.09); p = 0.027] (Figure 3) and on SGRQ activity domain [OR 1.45 (95% IC 1.04-2.03); p = 0.029]. In a second model, when BODE index was replaced by its variables (BMI, MMRC, FEV_1 _and 6MWD), Δ MMRC was the predictor of clinically significant worsening on SGRQ total score [OR 2.73 (95% IC 1.47-5.07); p = 0.001] (Figure 4) and on activity domain [OR 1.67 (95% IC 1.04-2.03); p = 0.031]. Predictor variables of clinically significant stability/improvement or worsening on SGRQ symptom and impact domains were not identified.

## Discussion

Results of this study showed that the BODE index was a predictor of HS at baseline and after three years. The components of BODE index associated with HS were dyspnea sensation and FEV_1_. The rate of exacerbations also influenced the HS overtime. Clinically significant deterioration of HS was associated with increase in dyspnea perception during the follow-up. These findings reinforce the importance of therapeutic measures to control the dyspnea, prevent progression of airflow obstruction and exacerbations as tools to maintain or improve the health status of COPD patients.

We observed a significant worsening in the activity domain and SGRQ total score during the follow-up. Our results are consistent with those of Oga et al. [7], who showed a deterioration of health status as indicated by increased activity and impact domains and SGRQ total scores after a five-year period. Besides the statistically significant deterioration of HS overtime, our results showed that 51% of the patients presented clinically significant worsening (≥ 4%) on SGRQ total score; 59% of these patients presented severe to very severe disease. Oga et al. [7] showed that the mean annual change in the health status scores was 1.87 units/year from the SGRQ total score and took 2.14 years to deteriorate by a clinically significant worsening of 4 units.

We observed that the SGRQ total scores tended to be higher in patients with more advanced disease according to GOLD staging system; however, we did not find differences when patients with moderate and severe disease were compared or between patients with severe and very severe disease. Hajiro et al. [24] also demonstrated that patients in the worst disease stage had the worst scores on SGRQ total score; in addition, GOLD staging of COPD was shown to be associated with important differences in health status between severe and moderate disease, but not between other disease stages [25]. Cross-sectional studies showed that BODE index is better correlated to health status as assessed by a disease-speciﬁc index for COPD than the GOLD staging criteria based largely on the FEV_1 _[26,27]. Ong et al. [26] evaluated 100 patients with stable COPD and found that important differences in health status between the highest classes (classes 3 and 4) of the BODE classiﬁcation system were observed but not between lower grade consecutive classes. In our study, we found that HS did not change between the classes 2, 3 e 4. Despite the small number of patients in class 4, this finding shows that the health status cannot be inferred from the BODE index and should be systematically assessed in the individual patient. Therefore, these studies show that there is not linearity of differences between SGRQ values in different stages of severity.

Our results showed that FEV_1 _was a predictor of HS after a three-year period. Lin et al. [11] showed that with the decrease of airflow limitation, SGRQ total and SGRQ subscales were increased correspondingly at baseline and the end of 1 year. However, in Oga et al. [7], the changes in health status assessed by the SGRQ total scores were weakly correlated with the changes in FEV_1_%.

In our study, dyspnea was strongly associated with HS at all times. The Transition Dyspnea Index (TDI) measures changes in dyspnea sensation from baseline over time; however, the patient has to recall their baseline (Baseline Dyspnea Index) in order to answer questions regarding the TDI [28]. Therefore, we used the MMRC scale which is a traditional instrument included in the BODE index [20]. In multiple logistic regression, when the BODE index was replaced by its variables, worsening of one unit in MMRC doubled the risk of worsening of the SGRQ total score. The association between dyspnea and HS is known from results of previous cross-sectional and longitudinal studies [7,9,29]. In a five year follow-up study, annual changes of the SGRQ total score showed correlation with changes in the dyspnea intensity, assessed by MMRC [7]. In the same study, the authors verified correlation of annual changes of SGRQ total score with anxiety, depression scores and peak oxygen uptake. However, the authors did not evaluate the influence of the BODE index and the number of exacerbation in the changes of health status.

Our results showed that exacerbation rate was associated with impairment of HS during follow-up. This finding reinforces the impact of exacerbation in clinical outcomes; exacerbations of COPD indicate clinical instability and progression of the disease and are associated with increased morbidity, deterioration of comorbidities, and reduced health status [12]. In our study, patients who had at least one exacerbation during follow-up presented with higher SGRQ scores at baseline when compared to patients without exacerbations. Spencer et al. [30] showed that baseline SGRQ scores were significantly higher in patients who experienced an exacerbation as compared to those without exacerbations during the three-year follow up. Miravitlles et al. [31] found that among patients with moderate COPD, those with frequent exacerbations had a greater change in SGRQ total score (2 units per year) than those with infrequent exacerbations, after controlling for baseline characteristics at 2 year follow-up. However, the number of exacerbation variables may have limitations, since Seemungal et al. [8] have shown that about 50% of exacerbations are untreated, or at least not reported to physicians.

In the multiple linear regression analysis, we verified that the BODE index was a predictor of health status overtime. In addition, worsening of one unit of the BODE index has a 50% increased risk of worsening in the SGRQ total score and activity domain. Our findings are in accord with Lin et al. [11], who found by multiple linear regression that the BODE index was associated with SGRQ at baseline at the end of 1 year follow up after adjustment for age, gender, and smoking status. COPD is a complex multidimensional disease and the BODE index, a multidimensional grading system, has been shown to be a superior predictor of the risk of death [20]. BODE index is also predictor of acute exacerbations [32], hospitalization [33] and health status [11]. However, it does not incorporate the exacerbation of COPD, which is an important outcome marker.

As shown in our study, HS impairment was associated with more than one outcome measure and may reflect the lung and systemic effects of COPD. Therefore, predictors of HS assessments will enable clinicians to evaluate the overall efficacy of the management of disease. Health-status as a concept of high complexity is assessed indirectly and requires the application of specially designed questionnaires [2]. The SGRQ has been widely used in clinical trials as an endpoint to assess the effects of treatment and management interventions on health status in COPD [34,35], although their use in clinical practice is hampered since this instrument is relatively time and resource consuming. Self-rated health (SRH) data may be an alternative because of their simplicity of collection and strong association with outcome [36]; such it has been shown that SRH predicted exacerbations and hospitalizations in patients with COPD [37]. In additional, SHR was associated with similar HS determinants as in present study [38-40]. However, nowadays the formal questionnaires can be completed in computers, in several places, and the scores can be easily obtained. We believe that both forms are necessary to be available to attend outpatients units with different resources.

There are some limitations in our study. We did not include depression and anxiety evaluations. In fact, psychological factors were shown to have an important impact in health status of COPD patients [41]. The lack of these evaluations in our study may have influenced the results and therefore, psychological or socio-cultural aspects should also be verified in further studies designed to evaluate the HS over time. In addition, patients came from the outpatient clinic of a university hospital and; therefore, may not represent the COPD population at large.

## Conclusions

In summary, HS deteriorated significantly over the three-year period and the predictors of HS do not change over time. BODE index and dyspnea were predictors at baseline and after three years. Exacerbation was also a predictor of HS after three years. These results suggest that health status scores should be included as part of a comprehensive assessment to evaluate disease progression.

## Abbreviations

6MWD: six-minute walk distance; BMI: Body mass index; BODE: BMI/airflow obstruction/dyspnea/exercise capacity; BTS: Brazilian Thoracic Society; COPD: Chronic obstructive pulmonary disease; FEV_1_: Forced expiratory volume in 1 second; FVC: Forced expiratory vital capacity; GOLD: Global initiative for chronic obstructive lung disease; HS: health status; MCID: Minimum clinically important difference; MMRC: Modified Medical Research Council; QoL: patients' quality of life; SGRQ: Saint George's Respiratory Questionnaire; SpO_2_: pulse oximetry; TDI: Transition Dyspnea Index.

## Competing interests

The authors declare that they have no competing interests.

## Authors' contributions

RF and IG conceptualized the study. SET carried out the statistical analyses; RF, SET and IG analyzed the data and drafted the manuscript. RF, LMOC and CRN obtained the data. All authors provided input on the interpretation and they read and approved of the final draft of the manuscript.

## Supplementary Material

Additional file 1 **Simple correlation analysis between baseline and final SGRQ score and studied variables**. Simple correlation analysis between baseline and final SGRQ score and age, gender, smoking, SpO_2_, BODE index or its components, and Charlson index. Click here for file
