# Supplementary material for: Predictors of health status do not change over three-year periods and exacerbation makes difference in chronic obstructive pulmonary disease
Source: Health Qual Life Outcomes. 2011 Dec 9;9:112. doi: 10.1186/1477-7525-9-112 (PMC3254128; doi:10.1186/1477-7525-9-112)
Supplement: Additional file 1 — Simple correlation analysis between baseline and final SGRQ score and studied variables. Simple correlation analysis between baseline and final SGRQ score and age, gender, smoking, SpO2, BODE index or its components, and Charlson index. [file 1477-7525-9-112-S1.DOC]

**Additional file 1.** Simple correlation analysis between baseline and final SGRQ score and studied variables

|  | Variables | Coefficient (r) | | p-Value |
| --- | --- | --- | --- | --- |
| Baseline total SGRQ | Age, years | | -0.29 | **0.004** |
|  | Gender, male | | -0.03 | **0.002** |
|  | Smoking status, yes | | 0.01 | 0.284 |
|  | SpO_2_, % | | - 0.2 | **0.011** |
|  | BODE, score | | 0.57 | **<0.001** |
|  | FEV_1_, % pred | | -0.34 | **<0.001** |
|  | MMRC, score | | 0.71 | **<0.001** |
|  | 6MWD, meters | | -0.27 | **0.007** |
|  | BMI, kg/m^2^ | | -0.02 | 0.791 |
|  | Charlson, score | | -0.02 | **0.014** |
|  |  | |  |  |
| Final total SGRQ | Age, years | | -0.09 | 0.358 |
|  | Gender, male | | - 0.18 | **0.042** |
|  | Smoking status, yes | | -0.01 | 0.646 |
|  | SpO_2_, % | | - 0.43 | **<0.001** |
|  | BODE, score | | 0.66 | **<0.001** |
|  | FEV_1_, % pred | | -0.52 | **<0.001** |
|  | MMRC, score | | 0.75 | **<0.001** |
|  | 6MWD, meters | | -0.38 | **<0.001** |
|  | BMI, kg/m^2^ | | 0.12 | 0.265 |
|  | Charlson, score | | -0.08 | 0.441 |
|  | Exacerbation | | 0.50 | **<0.001** |

SpO_2_: pulse oximetry; FEV_1_: forced expiratory volume in the first second (% of predicted); MMRC: Modified Medical Research Council**;** 6MWD: six-minute walking distance; BMI: body mass index; p<0.05.
